# Effects of airway obstruction and hyperinflation on electrocardiographic axes in COPD

**DOI:** 10.1186/s12931-019-1025-y

**Published:** 2019-03-27

**Authors:** Peter Alter, Henrik Watz, Kathrin Kahnert, Klaus F. Rabe, Frank Biertz, Ronald Fischer, Philip Jung, Jana Graf, Robert Bals, Claus F. Vogelmeier, Rudolf A. Jörres

**Affiliations:** 1 Department of Medicine, Pulmonary and Critical Care Medicine, Philipps University of Marburg (UMR), Member of the German Center for Lung Research (DZL), Baldingerstrasse 1, 35033 Marburg, Germany; 2 Pulmonary Research Institute at LungenClinic Grosshansdorf, Airway Research Center North (ARCN), Member of the German Center for Lung Research (DZL), Grosshansdorf, Germany; 3 Department of Internal Medicine V, Ludwig Maximilians University (LMU), Comprehensive Pneumology Center Munich (CPC-M), Member of the German Center for Lung Research (DZL), Munich, Germany; 4 Department of Internal Medicine, LungenClinic Grosshansdorf and Christian-Albrechts University, Kiel, Airway Research Center North (ARCN), Member of the German Center for Lung Research (DZL), Grosshansdorf, Germany; 5 Institute for Biostatistics, Center for Biometry, Medical Informatics and Medical Technology, Hannover Medical School, Hannover, Germany; 6 Internal Medicine, Dachau, Germany; 7 Institute and Outpatient Clinic for Occupational, Social and Environmental Medicine, Ludwig Maximilians University (LMU), Comprehensive Pneumology Center Munich (CPC-M), Member of the German Center for Lung Research (DZL), Ziemssenstrasse 1, 80336 Munich, Germany; 8 Department of Internal Medicine V - Pulmonology, Allergology, Intensive Care Medicine, Saarland University Hospital, Homburg, Germany

**Keywords:** COPD, Airway obstruction, Hyperinflation, Electrocardiographic axis, P wave axis, QRS axis, T wave axis

## Abstract

**Background:**

COPD influences cardiac function and morphology. Changes of the electrical heart axes have been largely attributed to a supposed increased right heart load in the past, whereas a potential involvement of the left heart has not been sufficiently addressed. It is not known to which extent these alterations are due to changes in lung function parameters. We therefore quantified the relationship between airway obstruction, lung hyperinflation, several echo- and electrocardiographic parameters on the orientation of the electrocardiographic (ECG) P, QRS and T wave axis in COPD.

**Methods:**

Data from the COPD cohort COSYCONET were analyzed, using forced expiratory volume in 1 s (FEV_1_), functional residual capacity (FRC), left ventricular (LV) mass, and ECG data.

**Results:**

One thousand, one hundred and ninety-five patients fulfilled the inclusion criteria (mean ± SD age: 63.9 ± 8.4 years; GOLD 0–4: 175/107/468/363/82). Left ventricular (LV) mass decreased from GOLD grades 1–4 (*p* = 0.002), whereas no differences in right ventricular wall thickness were observed. All three ECG axes were significantly associated with FEV_1_ and FRC. The QRS axes according to GOLD grades 0–4 were (mean ± SD): 26.2° ± 37.5°, 27.0° ± 37.7°, 31.7° ± 42.5°, 46.6° ± 42.2°, 47.4° ± 49.4°. Effects of lung function resulted in a clockwise rotation of the axes by 25°-30° in COPD with severe airway disease. There were additional associations with BMI, diastolic blood pressure, RR interval, QT duration and LV mass.

**Conclusion:**

Significant clockwise rotations of the electrical axes as a function of airway obstruction and lung hyperinflation were shown. The changes are likely to result from both a change of the anatomical orientation of the heart within the thoracic cavity and a reduced LV mass in COPD. The influences on the electrical axes reach an extent that could bias the ECG interpretation. The magnitude of lung function impairment should be taken into account to uncover other cardiac disease and to prevent misdiagnosis.

**Electronic supplementary material:**

The online version of this article (10.1186/s12931-019-1025-y) contains supplementary material, which is available to authorized users.

## Background

Cardiovascular comorbidities are common in patients with chronic obstructive pulmonary disease (COPD) [1–3]. This includes morphological and functional alterations of the heart. For example, the severity of COPD is known to be inversely related to left ventricular (LV) size and mass [4–6]. One of the basic diagnostic criteria for cardiac disorders is the definition of the electrical axes from the standard surface electrocardiogram (ECG) [7]. These are the P wave, QRS and T wave axes that can be obtained by established algorithms. The QRS axis is related to the spread of left and right ventricular (RV) depolarization, being dominated by the LV, since its muscular mass far exceeds that of the RV. A common alteration, for instance, is a counterclockwise leftward shift associated with LV hypertrophy resulting from hypertension. The P wave axis reflects atrial depolarization, with changes being suggestive of either left or right atrial predominance, and the T wave finally reflects ventricular repolarization. Due to alterations of the heart in COPD, changes in the orientation of the electrical axes are to be expected independent of or in addition to primary cardiac disease.

Verticalization of the P wave axis in COPD has been reported [8–10], as well as a positive correlation between the P wave vector and radiographic evidence of emphysema [11]. Increased heart rate is a common finding in COPD and linked to its severity and prognosis [12]. Associated changes of de- and repolarization may also interfere with the orientation of the axes. Additionally, the mechanical environment of the heart is likely to be altered by lung hyperinflation and changes in intrathoracic pressures due to airway obstruction, also potentially exert influences. However, it is unclear how changes in the different lung function measures correlate with the magnitude of this effect, and whether the various types of axes are impacted differently. Such data are of clinical interest, as alterations in the electrical axes resulting purely from changes in lung function might bias the cardiologic diagnostic interpretation.

We therefore hypothesized that the electrical axes of the heart are related to lung function in patients with COPD. Airway obstruction and hyperinflation were evaluated as numerical predictors of the electrical heart axes.

## Methods

### Study cohort and participants

The study was performed using a subset of the baseline data of the German COPD cohort COSYCONET, which is a prospective, observational, multi-center cohort study in patients with stable COPD that aims to evaluate the role of comorbidities [13–15], including the relationship between lung and cardiovascular disease by ECG analysis and echocardiography [16, 17]. All study participants gave their written informed consent. The criteria of airflow limitation proposed by the Global Initiative for Obstructive Lung Disease (GOLD) [18] were applied to define spirometric GOLD grades 1–4.

For the present analysis, we used data from the recruitment phase and excluded patients with more than moderate heart valve disease, heart valve replacement, or other cardiac devices such as pacemakers/cardioverter-defibrillators. The analysis was restricted to patients with sinus rhythm and several criteria of completeness and plausibility of lung function, echocardiographic and ECG data were applied (see Additional file 1: Methods and Figure E1) [16, 17].

### Assessments

Spirometry and body plethysmography were performed following the recommendations of the American Thoracic Society (ATS)/European Respiratory Society (ERS) [19] and Deutsche Gesellschaft für Pneumologie und Beatmungsmedizin (DGP) [20–23], after inhalation of 400 μg salbutamol and 80 μg ipratropium bromide [13]. As a measure of lung hyperinflation, we chose functional residual capacity (FRC_pleth_; intra-thoracic gas volume, ITGV), the residual volume (RV), total lung capacity (TLC), and their ratio RV/TLC and forced expiratory volume in 1 s (FEV_1_) for airway obstruction. The diffusing capacity for carbon monoxide (TLCO) was determined via duplicate assessments of the single-breath method, and the transfer coefficient (KCO) as ratio of TLCO and alveolar volume (VA). Echocardiography was performed as recommended by the American Society of Echocardiography and the European Association of Cardiovascular Imaging [24]. The assessments included the left ventricular end-diastolic and end-systolic diameter (LVEDD, LVESD), LV mass and the right ventricular (RV) wall thickness as indicator of RV hypertrophy as well as heart rate lowering medication. Besides the electrical axes, we selected the ECG derived RR interval as measure of heart rate, and QT duration as measure of repolarization. Standard ECG were obtained and analyzed using the recorder EL10 (VERITAS™, 9515–001-50-ENG REV A1, Mortara Instruments, Inc., Milwaukee, Wisconsin, USA).

### Data analysis

FEV_1_ and FRC were evaluated as percent predicted values [25–27]. Cardiac size was expressed as LV mass normalized to body surface area [g/m^2^]. The RR interval was obtained as the mean of 10.88 ± 2.08 (mean ± SD) consecutive QRS complexes. The QT duration was used as measured, i.e. without heart rate correction, since heart rate was considered as distinct parameter.

For descriptive purposes mean values and standard deviations (SD) or standard errors of the mean (SE) were computed. Differences between groups were evaluated via analysis of variance (ANOVA) and by Tukey-HSD post-hoc comparisons. Univariate multiple linear regression analyses were employed to determine the influences of sex, age and medication on the different variables. Variables were adjusted for these three influencing factors via computation of non-standardized residuals and used for further analyses. Multivariate multiple linear regression analyses were used to determine the associations between FEV_1_ % predicted, FRC % predicted, BMI and diastolic blood pressure as predictors, and LV mass, RR interval, QT duration, P wave axis, QRS axis and T wave axis as dependent variables. For all estimates of regression coefficients, 95% confidence intervals were computed.

To disentangle the multiple relationships between the measured variables, structural equation modelling (SEM) was employed [14, 16, 17, 28, 29]. The construct named “ECG axes” comprised the P wave, QRS and T wave axes. The goodness of fit was evaluated by the comparative fit index (CFI) and the root mean square error of approximation (RMSEA). Chi-square data are also given. For all computations the software IBM SPSS Statistics 24.0.0.1 and Amos 24.0.0 (Wexford, PA, USA) was used. Statistical significance was assumed for *p* < 0.05.

## Results

### Study population

A total of 1195 stable COPD patients were analyzed. The selection process of the cohort is depicted in Additional file 1: Figure E1, and baseline characteristics are shown in Table 1. LV mass decreased significantly from GOLD grades 1–4 (mean ± SD: 111.5 ± 34.0, 109.5 ± 34.1, 103.0 ± 36.1, 97.6 ± 34.9 g/m^2^; *p* = 0.002), whereas no differences in RV wall thickness were observed (mean ± SD: 6.2 ± 6.1, 5.7 ± 3.3, 5.9 ± 2.3, 6.3 ± 4.4 mm).

**Table 1:** Baseline characteristics of the study cohort (*n* = 1195)

Parameter	Mean values ± SD or numbers
Anthropometry	
Age, years	63.9 [±8.4]
Sex, m/f	677/518
BMI, kg/m^2^	26.7 [±5.0]
Diastolic blood pressure, mmHg	75.0 [±10.4]
Lung function	
GOLD 0/1/2/3/4	175/107/468/363/82
FEV_1_ % predicted	58.8 [±20.7]
FRC % predicted	145.0 [±34.9]
RV, l	3.77 [±1.15]
TLC, l	7.16 [±1.46]
RV/TLC	0.52 [±0.11]
TLCO % predicted	59.9 [±22.8]
KCO % predicted	67.5 [±22.9]
Echocardiographic measures	
LVEDD, mm	47.5 [±6.5]
LVESD, mm	31.2 [±6.6]
LV mass, g/m^2^	106.6 [±34.5]
RV wall thickness, mm	5.9 [±3.5]
Electrocardiogram	
RR interval, ms	847.8 [±137.2]
QT duration, ms	386.3 [±29.5]
P wave axis, degree	60.5 [±25.0]
QRS axis, degree	36.1 [±42.6]
T wave axis, degree	53.3 [±23.1]

The table shows mean values [± standard deviations], except for gender and GOLD grade. BMI = body-mass index. Lung function: FEV_1_ = forced expiratory volume in 1 s, FRC = functional residual capacity, RV = residual volume; TLC = total lung capacity; TLCO = transfer factor of carbon monoxide (CO); KCO = CO transfer coefficient (ratio of TLCO and alveolar volume). Echocardiographic measures: LV = left ventricular; LVEDD = left ventricular end-diastolic diameter; LVESD = left ventricular end-systolic diameter; RV = right ventricular

### Electrical axes as related to GOLD grades

When averaged over the whole study population, the orientations of P wave, QRS and T wave axes differed significantly from each other (mean ± SD: 60.5° ± 25.0°, 36.1° ± 42.6°, 53.3° ± 23.1°, respectively; repeated-measures by ANOVA and Bonferroni-corrected comparisons, *p* < 0.001 for each pairwise comparison).

**Fig. 1:**
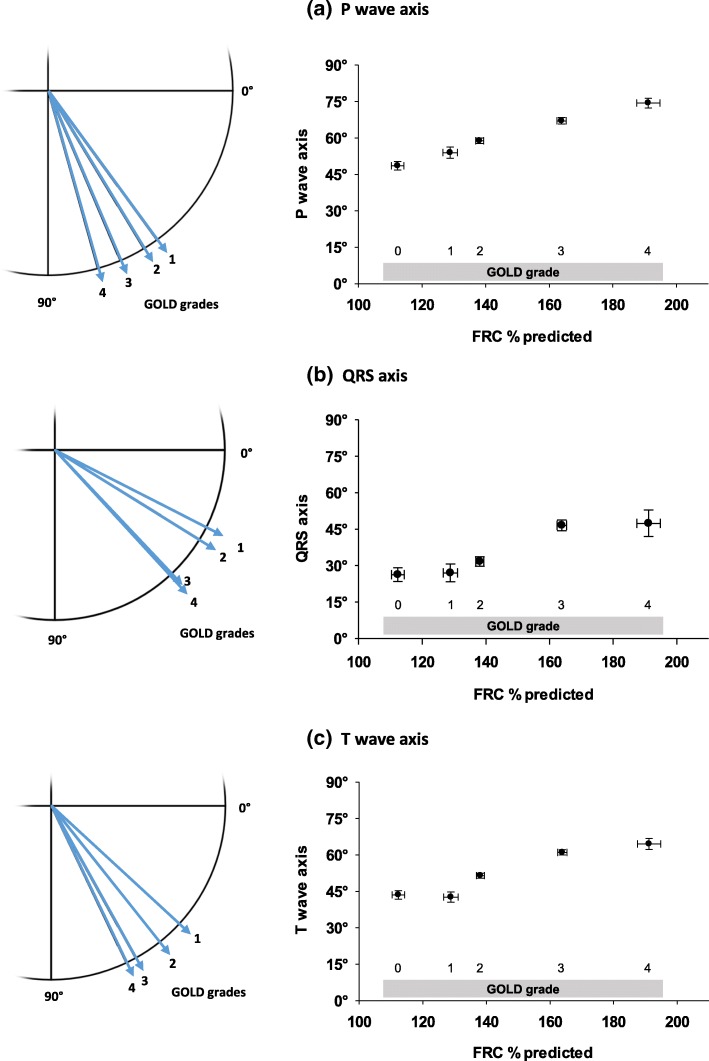
Mean values of the orientations of P wave (**a**), QRS (**b**) and T wave axes (**c**) using the Cabrera format are shown for spirometric GOLD grades 1–4 (left panel). GOLD grade 0 axes did not differ significantly from GOLD 1 and were thus omitted in the illustration to prevent an overlay. To show the additional dependence of the axes on FRC, plots of mean values versus mean values of FRC % predicted and the standard error of mean (bidirectional) for each GOLD grade 0–1 is shown (right panel). Post hoc comparisons revealed multiple significant differences of the axis orientation among GOLD grades as indicated by the means and error bars. In particular, significant differences were observed for all axes between GOLD grade 1 and 3 (*p* < 0.001), GOLD 1 and 4 (*p* < 0.001; except QRS: *p* = 0.008), GOLD grade 2 and 3 (*p* < 0.001), GOLD 2 and 4 (*p* < 0.001; except QRS: *p* = 0.015)

The mean orientation of the P wave axis according to the spirometric GOLD grades 0–4 is illustrated in the left panel of Fig. 1a, while the right panel shows the values plotted against mean values of FRC % predicted observed for each GOLD grade. The rotation of the P wave axis significantly increased across the GOLD grades (*p* < 0.001). Pairwise post hoc comparisons of the axis orientations between GOLD grades revealed significant (*p* < 0.05 each) differences, except between grade 0 and 1 and between grade 1 and 2.

In a similar manner, mean QRS axes are illustrated in Fig. 1b. Again, values significantly differed across GOLD grades (*p* < 0.001). There was a clear trend towards an increased clockwise rotation in more severe airflow limitation. Post hoc comparisons revealed significant (*p* < 0.05 each) differences between a disease severity not exceeding moderate grades (GOLD 0 to 2) compared with severe to very severe COPD (GOLD 3 and 4). The relationship of the QRS orientation to FRC % predicted across GOLD grades is illustrated.

The results for the mean T wave axis are analogously shown in Fig. 1c, with a significant difference across all GOLD grades (*p* < 0.001). There were significant (*p* < 0.05 each) differences between all GOLD grades, except between grade 0 and 1 and between grade 3 and 4. Again, the relationship to the mean values of FRC % predicted for the different GOLD grades is shown.

### Changes of the electrical axes due to the extent of lung function impairment

**Fig. 2:**
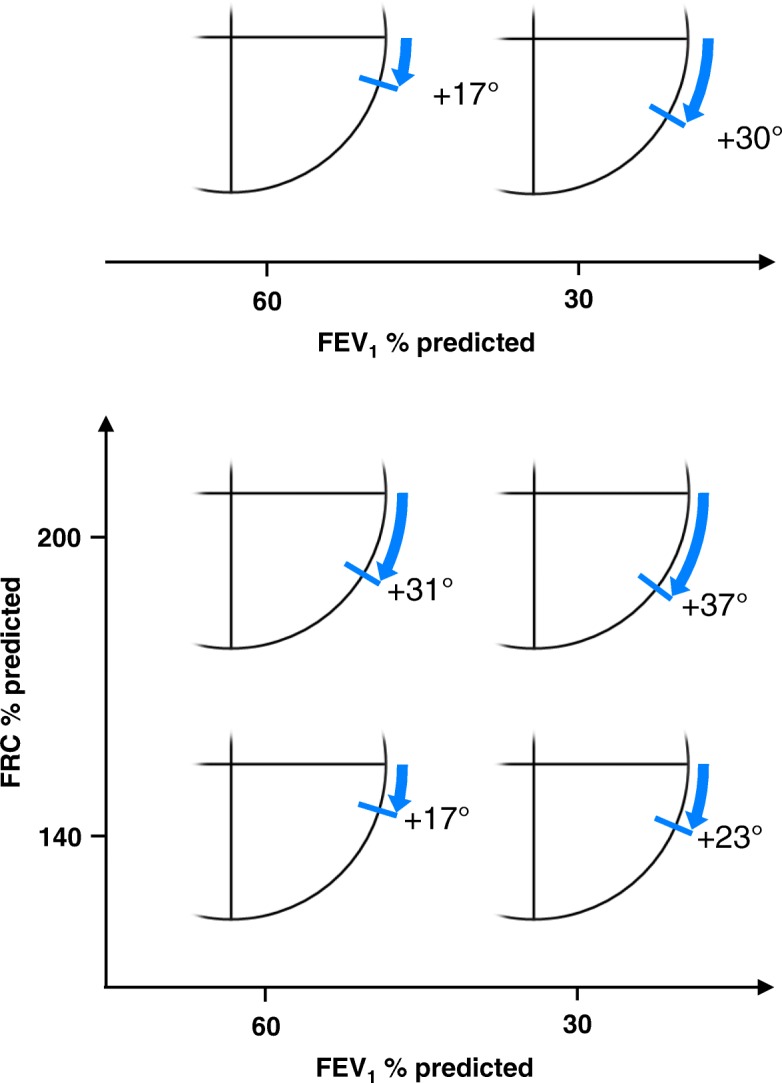
Upper panel: Estimated incremental clockwise rotation of the QRS axis based on FEV_1_ in univariate regression analysis (see Additional file 1: Table E1) for mild or severe airway obstruction (FEV_1_ 60 or 30% predicted, GLI). Lower panel: Estimated incremental clockwise rotation of the QRS axis based on bivariate regression analysis taking into account both FEV_1_ and FRC (see Additional file 1: Table E1). The circle segments show the estimated effects of lung function on the electrical rightward rotation for four combinations of mild or severe obstruction (FEV_1_ 60 or 30% predicted, GLI) with mild or severe hyperinflation (FRC 140 or 200% predicted, ECSC)

We assessed the magnitude of the relationship between ECG axes and lung function using multivariate multiple linear regression analysis, with the three ECG axes as dependent variables against FEV_1_ % predicted and FRC % predicted as covariates. In accordance with the GOLD definition of COPD [18], this subanalysis was purely restricted to GOLD grades 1–4 (*n* = 1020). Additional file 1: Table E1 shows regression coefficients of FEV_1_ and FRC as predictors of the electrical axes. Since both predictors are cross-linked with each other and FRC is not always available in clinical practice, the analysis was rerun using FEV_1_ as predictor only. The estimated incremental rotation of the QRS axis as a function of FEV_1_ (univariate analysis) and as function of both FEV_1_ and FRC (bivariate analysis) is illustrated in Fig. 2. This analysis demonstrates that airway obstruction and hyperinflation are significant predictors of the electrical axes (for regression analyses including the P and T wave axis see Additional file 1: Figure E2).

The measured distribution of the QRS axis across standard sectors is shown in Additional file 1: Figure E3. It is noteworthy that when influences of FEV_1_ and FRC were subtracted, the distribution of the QRS axes shifts from a vertical type (sector 60° to 90°, upper panel) to a normal (sector 30° to 60°) as the most frequent type (lower panel).

### Adjustment for sex, age and medication

To account for possible effects of confounders on measured variables, we also evaluated their relationship to sex, age and heart rate-lowering medication using univariate multiple linear regression analyses. All parameters showed a significant dependence on sex except FEV_1_ % predicted and diastolic blood pressure, whereas age was significantly associated with FEV_1_ and FRC % predicted, diastolic blood pressure, LV mass and QRS and T wave axis. Heart rate lowering medication (including betablockers, verapamil-type calcium channel blockers [phenylalkylamines] and ivabradine), was significantly related only to FEV_1_ and FRC % predicted (*p* < 0.05 each). In all following analyses we used the values that were adjusted for sex, age and medication according to these results.

### Effects of lung function, LV mass, RR interval and QT duration on the electrical axes

The relationship between the selected ECG and echocardiographic LV mass as dependent variables, and FEV_1_ % predicted, FRC % predicted, BMI and diastolic blood pressure as covariates was determined by multivariate multiple linear regression analysis. FEV_1_ % predicted was correlated with the RR interval, the QT duration and all three electrical axes. FRC % predicted correlated with the RR interval, QT duration and the three axes. BMI was associated with all dependent variables, with the exception of QT duration. Diastolic blood pressure correlated with all variables except LV mass and the T wave axis (Additional file 1: Table E2).

### Comprehensive structural equation modelling

**Fig. 3:**
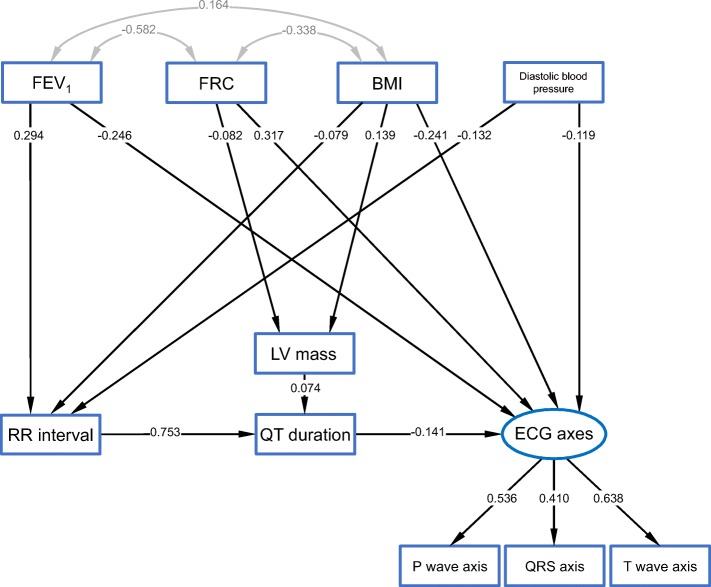
Structural equation model (SEM) providing a comprehensive description of the multiple relationships between influencing factors (top) and dependent variables (below). All measured (manifest) variables are indicated by rectangles. A latent variable (indicated by an oval) named “ECG axes” with the indicator variables P wave, QRS and T wave axes could be constructed in order to summarize the axes orientation and their fixed relationship to each other into a single variable. The lines with one arrow describe unidirectional effects, standardized regression coefficients are given; those with two arrows indicate mutual dependences in terms of correlations, correlation coefficients are given. The error terms needed for mathematical reasons for all dependent variables (i.e. all at which a unidirectional arrow ends) have been omitted for the sake of clarity. The numerical values of the respective unstandardized regression coefficients and covariances coefficients as well as measures of statistical significance are given in Additional file 1: Table E3

Given these multiple interdependences between parameters, we aimed to determine their relative importance in a network of associations via SEM, which is an extension of multiple regression and factor analysis [14, 16]. The SEM that showed the best fit and which represented a consistent and interpretable network of relationships is shown in Fig. 3; the estimates of the respective regression coefficients and covariances are given in Additional file 1: Table E3. The model comprised a latent variable named “ECG axes” which summarizes the information from the P wave, QRS and T wave axis. Although the mean values of the QRS axis were different from those of the P and T wave axes (Fig. 1), they could be summarized in one latent variable, since all of them were highly correlated with each other and depended in a similar manner on the covariates. LV size was represented by LV mass, which was related to the QT duration. The RR interval was connected to the QT duration, and this was connected to the ECG axes. This pattern of relationships fitted the data very well which was confirmed by the high values of critical ratios in Additional file 1: Table E3. The model showed a chi-squared value of 45.5, with 27 degrees of freedom (*p* = 0.014); the CFI was 0.992, with an RMSEA of 0.024 (90%CI 0.011; 0.036), which indicates an acceptable model that does not significantly deviate from the data. A detailed sensitivity analysis is given in Additional file 1: Results.

## Discussion

The present study demonstrates significant associations of the degree of airway obstruction and lung hyperinflation with the orientation of the electrocardiographic heart axes in patients with COPD. The association comprised direct influences of both FEV_1_, a measure of airway obstruction, and FRC, a measure of lung hyperinflation, but there were also indirect influences that were mediated through associations with other variables, including LV mass, the RR interval and the QT duration. This network of relationships was studied by using structural equation modelling as a statistical method designed to describe such networks. These relationships appear to be plausible from a pathophysiological point of view. Besides well-known qualitative influences of lung disease on the electrical heart axes, the present study for the first time quantifies influences of the magnitude of lung function impairment.

Determination of the QRS axis is a basic diagnostic criterion that is commonly used clinically to gain evidence, e.g. for LV hypertrophy, but also for an increased right heart load, e.g. due to pulmonary hypertension or pulmonary embolism. The large clockwise rotations of about 25 degrees on average significantly affect the judgement of the electrical type. This helps to uncover other cardiac disease and to prevent misdiagnosis, which is particularly valuable as on the one hand patients with COPD often have cardiac disease, but there are also significant numbers of individuals without such concomitant disorders [30]. For instance, assuming a patient who developed LV hypertrophy as consequence of long-term hypertension. Usually, a left-axis deviation of the QRS complex could be expected. Concomitant COPD can lead to a shift of the vector into the normal range, and thus presence of hypertrophy could be masked. Vice versa, also presence of COPD contributing to an incremental clockwise rotation could be overlooked, when allegedly normal values were found. The present study allows a numerical correction of the measured axis for influences of lung function, univariate based on FEV_1_ only and bivariate based on both FEV_1_ and FRC.

It is conceivable that lung hyperinflation affects the anatomical axis of the heart mechanically within the thoracic cavity, and consequently the electrical axes. An interesting finding was that airway obstruction in terms of FEV_1_ also played a role despite the fact that a decrease of FEV_1_ and consecutive increases in FRC are generally related to each other; i.e. an increase in FRC may be due to expiratory flow limitation during tidal breathing in dynamic hyperinflation or reduced elastic recoil in static hyperinflation. Both mechanisms may not be strictly related to FEV_1_ but may affect heart function, e.g. by a reduced venous return due to increased thoracic and gastric pressure [31] and by an impaired transpulmonary flow in emphysema [4]. Indeed, based on z-scores, 948 out of 1195 participants (79.3%) were below the lower limit of normal (LLN) of TLCO, and only 247 equal or above.

Interestingly, the two lung function parameters worked in parallel on the ECG axes, but were to some extent counteracted by those of BMI, which was correlated with both FRC and FEV_1_. Therefore, it can be hypothesized that patients with high FRC and low FEV_1_ would demonstrate particularly strong effects on the rotations of the electrical axes if they also have a low BMI, e.g. in cachectic patients with pulmonary emphysema. It seems noteworthy that the direct influences of FRC and FEV_1_ on LV mass indicated a cardiac response to hyperinflation, which was linked to the QT duration that was also affected via the RR interval. Since the QRS axis depends on the electrical depolarization of both ventricles, one could argue that potential changes of the RV may have affected the findings. However, this appears unlikely, since no differences of the echocardiographic RV wall diameter or RV function was observed among GOLD grades. Moreover, the contributory extent of the RV to the QRS axis appears minor than that of the LV due to the much less RV mass.

Thus, we suggest that a superimposition of several effects rather than one sole dominator was responsible for the observed deviation of axes due to lung function. The regression coefficients suggest that the direct effects from FEV_1_, FRC, and BMI on the axes were dominant over indirect effects as mediated via interposed variables (SEM, Fig. 3). For quantification, the respective coefficients of the cascade of correlations (Additional file 1: Table E3) can be multiplied.

In the analyses using unadjusted values, there were significant differences among the average orientation of the three electrical axes. Moreover, there was a strong dependence of the axes on spirometric GOLD grades. Different slopes in the correlations of atrial and ventricular axes with lung function were observed. The QRS axis showed a stronger correlation with FEV_1_ and FRC than the P wave axis did, which can be seen in the regression coefficients (Additional file 1: Table E1). The T wave coefficient, indicating ventricular repolarization, is near to the ventricular QRS, which is not unexpected. Greater influences of lung function on the ventricular than on the atrial axis became also apparent when using FEV_1_ as predictor only (Fig. 3). This may result from a decrease of LV mass and/or size in increased COPD severity. Whether this truly reflects different mechanical effects or different phenotypes of COPD in terms of bronchitis and emphysema, cannot be determined from our data. In addition, morphological changes of the RV could interfere with the QRS and T wave axis.

## Limitations

Due to potential difficulties in obtaining echocardiography in patients with hyperinflation, meticulous criteria on plausibility and completeness were applied, which is reflected in the selection process and resulted in this subset of COSYCONET. Significant clockwise rotations of the electrical heart axes as a function of airway obstruction and lung hyperinflation were shown. It is likely that the observed changes result from both a rotation of the heart within the thoracic cavity and a reduced LV mass in COPD. Therefore, it would be worth knowing whether these findings on the electrical rotation were paralleled by a rotation of the anatomical heart axis, e.g. as assessable by cardiac computed tomography or magnetic resonance imaging. However, these data were not available for the examined cohort. Nevertheless, assessment of the electrical heart type based on the surface ECG is the diagnostic standard procedure, and considering quantitative influences of lung function is crucial for its accurate interpretation.

## Conclusions

The present study shows significant clockwise rotations of the electrical heart axes as a function of both airway obstruction and lung hyperinflation. Besides these direct effects, intermediate factors such as LV mass, heart rate and QT duration, were quantified. Lung function impairment affected the P wave, QRS and T wave axis in the same clockwise direction, which is compatible with a rotation of the heart within the thoracic cavity. Moreover, the degree of rotation was greater for the ventricular QRS and T wave axis than for the atrial P wave axis, which indicates a differential response. The decrease of LV mass, which is correlated with COPD severity, appears to contribute to the ventricular QRS axis rotation. These influences on the electrical axes reach an extent that could bias the interpretation of the ECG in severe COPD. Since assessment of the electrical heart axes based on the surface ECG is a diagnostic standard procedure, the magnitude of lung function impairment should be taken into account on a numerical basis in order to prevent misdiagnosis in concomitant cardiac and pulmonary disease.

## Additional files

Additional file 1: Supplementary methods, results and discussion. (DOCX 225 kb)

## Abbreviations

CFI: Comparative fit index
COPD: Chronic obstructive pulmonary disease
ECG: Electrocardiogram
FEV_1_: Forced expiratory volume in 1 s
FRC: Functional residual capacity by bodyplethysmography (FRC_pleth_; intra-thoracic gas volume, ITGV)
GOLD: Global Initiative for Obstructive Lung Disease
KCO: Carbon monoxide (CO) transfer coefficient (ratio of TLCO and alveolar volume)
LV: Left ventricle/ventricular (by echocardiography)
LVEDD: Left ventricular end-diastolic diameter
LVESD: Left ventricular end-systolic diameter
RMSEA: Root mean square error of approximation
RV: Right ventricle/ventricular (by echocardiography)
RV/TLC: Residual volume to total lung capacity ratio (by bodyplethysmography)
SEM: Structural equation modelling
TLCO: Transfer factor of carbon monoxide (CO)
