# Supplementary material for: Effects of airway obstruction and hyperinflation on electrocardiographic axes in COPD
Source: Respir Res. 2019 Mar 27;20:61. doi: 10.1186/s12931-019-1025-y (PMC6437876; doi:10.1186/s12931-019-1025-y)
Supplement: Supplementary file 1 — Supplementary methods, results and discussion. (DOCX 225 kb) [file 12931_2019_1025_MOESM1_ESM.docx]

**Effects of airway obstruction and hyperinflation on electrocardiographic axes in COPD**

Peter Alter, Prof, MD; Henrik Watz, MD; Kathrin Kahnert, MD; Klaus F. Rabe, Prof, MD, PhD; Frank Biertz; Ronald Fischer; Philip Jung, MD; Jana Graf; Robert Bals, Prof, MD, PhD;
Claus F. Vogelmeier, Prof, MD; Rudolf A. Jörres, PhD

**Online Data Supplement**

# Methods

COSYCONET is a prospective, observational, multi-center cohort study in patients with stable COPD. Inclusion and exclusion criteria for COSYCONET have been described previously [1]. All patients were at least 40 years of age, and had a diagnosis of COPD [2] or chronic bronchitis. To recruit as broad a population as possible, there were minimal exclusion criteria, none of which was cardiac in nature

For the present analysis, several criteria of completeness and plausibility of lung function and echocardiographic data [3, 4] were applied. We restricted the analysis to patients with values of LV mass ≤220 g/m² and with an FRC ≤250% predicted. These criteria were applied to avoid the potential effect of outliers on parametric statistical estimates. As the analyses require the reliable assessment of heart rate and of the spread of electrical conduction as reflected in the different ECG waves, 74 patients without ECG-documented sinus rhythm were also excluded. Moreover, 7 patients with values of BMI >45 kg/m² and 4 with diastolic blood pressure >110 mmHg were excluded, as such extreme values may have unpredictable effects on cardiac morphology and thus electrical conduction [5]. A total of 90 patients with an RR interval of >1250 ms, corresponding to a heart rate <48/min, or with an ST duration >500 ms, or values of P wave axis <-60° or >150°, or of QRS axis <-90° or >120°, or of T wave axis <-60° or >150° (all related to the standard leftward Cabrera reference axis) were also excluded, either because these values were regarded as un-physiological or because they represented outliers, with potential negative effect on the statistical parametric estimations (Figure E1).

The criteria of airflow limitation proposed by the Global Initiative for Obstructive Lung Disease (GOLD) [2] were applied to define spirometric GOLD grades 1 to 4 based on the impairment of forced expiratory volume in 1 s (FEV_1_) and requiring a ratio of FEV_1_ to forced vital capacity (FVC) of <0.7. Patients with FEV_1_/FVC above 0.7 but reporting symptoms of chronic bronchitis were summarized into a category ”COPD at risk”, the former GOLD grade 0. Within COSYCONET, all medication taken by the patients is recorded. From these data, the intake of heart rate-lowering drugs (all types of beta-blockers, verapamil-type calcium channel blockers [phenylalkylamines], and ivabradine) was evaluated and summarized into a variable indicating the presence or absence of such medication in each individual patient.

## Selection of variables for analysis

The selection of variables was based on pathophysiological considerations but also on exploratory data analyses that helped to identify redundant variables. The aim was to cover the three ECG axes, their most important influencing factors and other ECG and echocardiographic parameters that could be sensibly related to the axes. The guiding principle was a comprehensive description that avoided numerical instability due to collinearity of variables as well as large sets of variables that were too complex to be interpreted. As a measure of lung hyperinflation, we chose functional residual capacity (FRC), and FEV_1_ as a measure of airway obstruction. Furthermore, in the model we included body-mass index (BMI), which can influence the orientation of the axes, and diastolic blood pressure, which is known to be related to morphological as well as electrocardiographic characteristics. These variables were considered as independent influencing factors, in addition to age, sex, and the presence of heart-rate lowering medication. Besides the electrical axes that were the target of the study, we selected LVEDD, the left-ventricular end-systolic diameter (LVESD) and the derived parameter LV mass as indicators of LV size, the right ventricular (RV) wall thickness as indicator of RV hypertrophy, the ECG derived RR interval as measure of heart rate, and QT duration as measure of repolarization.

## Data analysis

We constructed a number of consecutive structural equation models (SEM), based on the results of the previous regression analyses, on additional factor analyses that evaluated the correlations between variables, and on pathophysiological considerations regarding known facts about variables that are related to each other. We incorporated both directly measured (manifest) variables and a construct (latent variable) built from measured variables (indicator variables) in order to condense the information from several indicators. The construct “ECG axes” comprised the P wave, QRS and T wave axes. In the majority of the SEM analyses, values adjusted for sex, age and heart-rate lowering medication, were used.

Several sensitivity analyses were applied to validate und confirm the model structure. First, the final SEM was re-run with crude, non-adjusted values, and, second, with values adjusted for sex and age only. Third, latter values were also used to define a model to which the binary variable “medication” was separately added in order to reveal which variables would be sensitive to medication. Fourth, instead of adjusted values of FEV_1_ % predicted and FRC % predicted, the corresponding z-scores were introduced into the final model, yielding concordant results. All of these findings underline the robustness of the SEM. For quantification of the SEM we used the maximum likelihood estimation procedure. To determine whether the results were robust we checked them via the generalized least squares method or the asymptotically distribution-free estimation method, both of which have weaker assumptions. The results were always concordant and confirm the model structure; details are therefore not reported.

# Results

## Figure E1. Flow diagram comprising the selection of participants included in the analysis


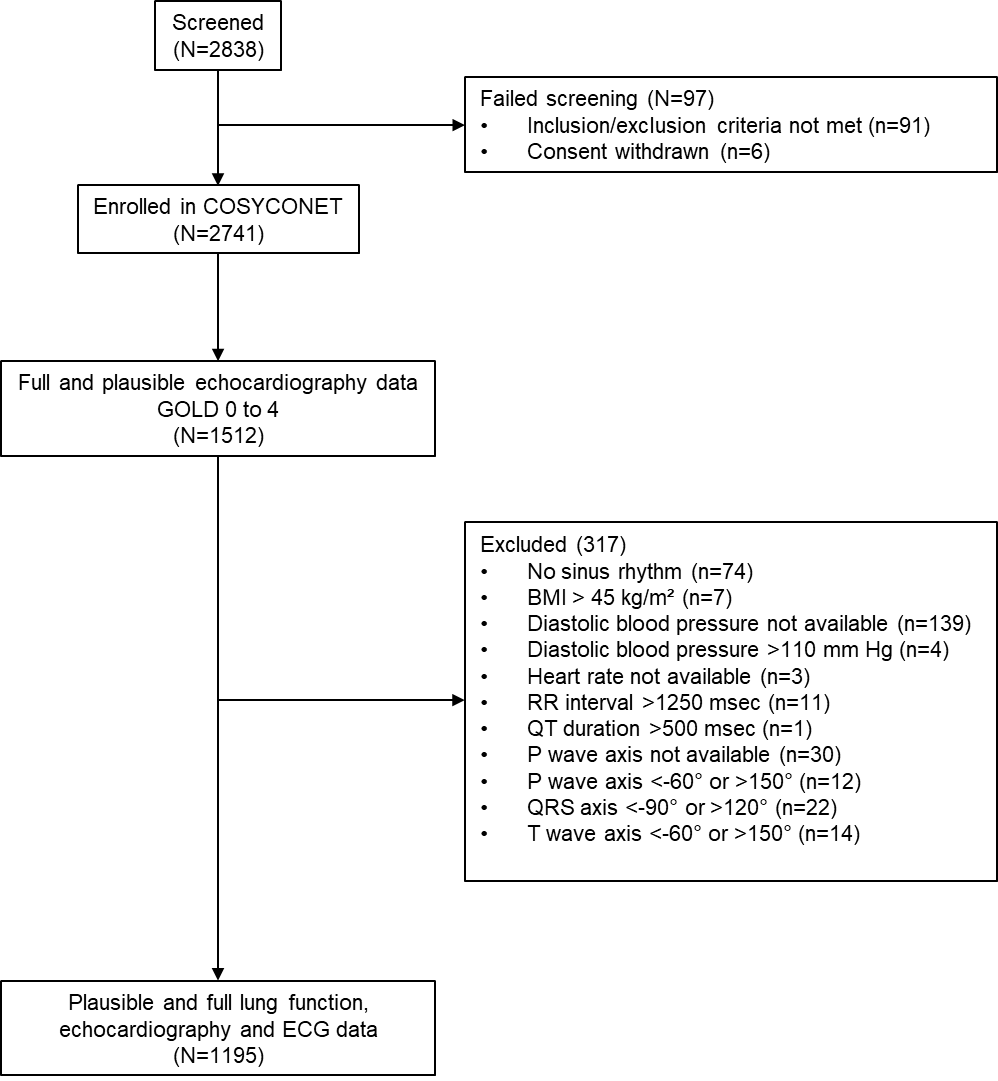


## Figure E2. Predicted clockwise rotation of ECG axes as a function of FEV_1_ % predicted

Predicted clockwise rotation of ECG axes as a function of FEV_1_ % predicted (univariate, results given in the lower panel of Table E1). This graph can be used to estimate the effect of lung function on axis orientations if only FEV_1_ is available as lung function parameter. The prediction based on both FEV_1_ and FRC (not illustrated, results given in the upper panel of Table E1) yields similar values when inserting typical observed combinations of FEV_1_ and FRC.

## Figure E3. Distribution of QRS axes

##
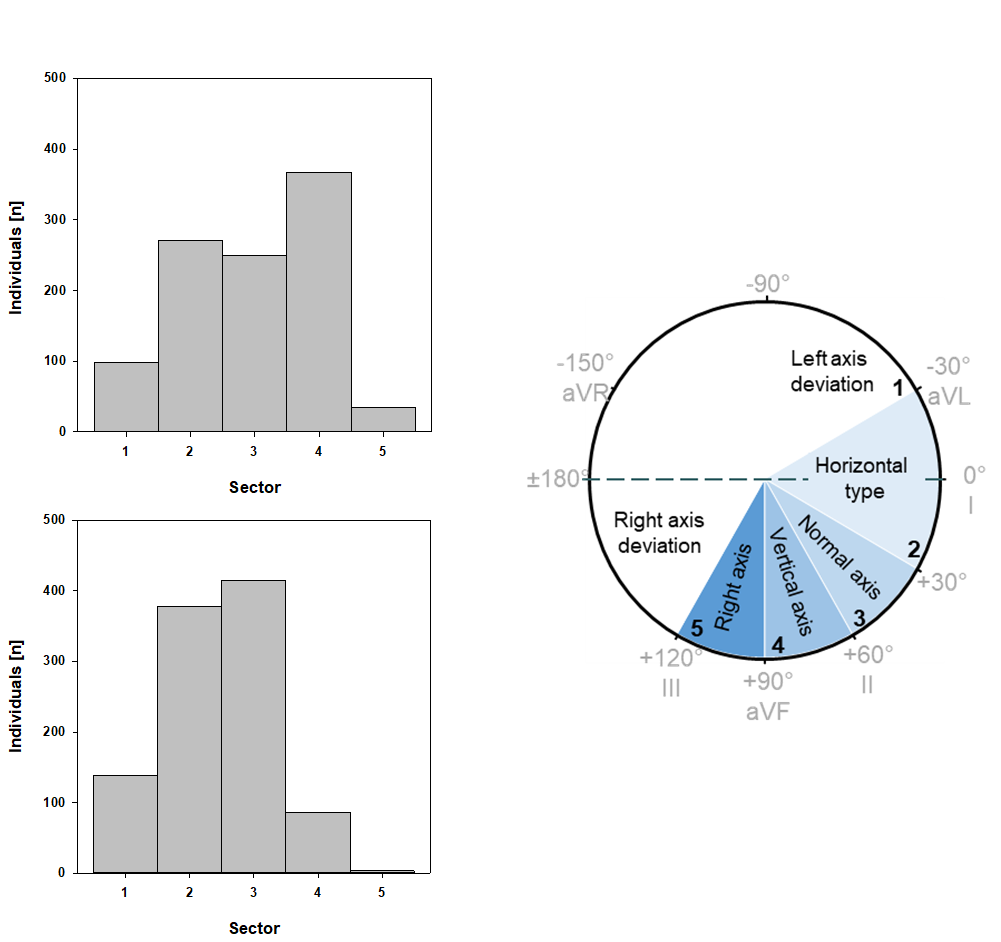


The figure illustrates the clockwise (rightward) shift of the distribution of QRS axis through the influence of lung function. It shows the histogram of the distribution of individuals across common types of QRS axis as directly measured (upper panel) and after subtraction of the influences of both FEV_1_ % predicted and FRC % predicted by regression analysis (lower panel) as shown in Table E1 (lower panel). Axes sectors (indicated 1 to 5) are defined in usual manner (right panel). The adjustment for lung function was based on the regression coefficients given in the upper panel of Table E1.

**Table E1. Association of lung function with electrical axes**

|  | Dependent variables | | |
| --- | --- | --- | --- |
|  | **P wave axis**  **degree** | **QRS axis**  **degree** | **T wave axis**  **degree** |
| Bivariate analysis using both FEV_1_ and FRC as predictors |  |  |  |
| FEV_1_ % predicted | 1.47  [0.52; 2.42] | 1.86  [0.17; 3.55] | 1.96  [1.11; 2.81] |
| FRC % predicted | 1.57  [1.04; 2.10] | 2.37  [1.43; 3.31] | 1.68  [1.21; 2.16] |
| Univariate analysis using  FEV_1_ as predictor |  |  |  |
| FEV_1_ % predicted | 3.04  [2.24; 3.84] | 4.23  [2.81; 5.65] | 3.64  [2.92; 4.37] |

Non-standardized univariate linear regression coefficients of the ECG axes versus lung function. To fulfill the pure definition of COPD, only patients of GOLD grades 1-4 were included in this part of the analysis.. Non-adjusted values were used for this analysis. The regression coefficients indicate the rightward rotation of each axis corresponding to a 10% unit decrease of FEV_1_ or increase of FRC % predicted, respectively. The upper two lines refer to a multiple regression model containing both FEV_1_ and FRC, with the estimated changes in the axes obtained by addition of the two contributions. The lower line refers to a regression analysis in which FEV_1_ is the only predictor. The numbers in the brackets represent the 95% confidence intervals. None of these intervals included zero, therefore all dependences were statistically significant (p<0.05). Abbreviations: FEV_1_ = forced expiratory volume in 1 second, FRC = functional residual capacity.

**Table E2. Results of multivariate linear regression analysis against the four covariates**

| **Dependent variables** | **Predictors** | | | |
| --- | --- | --- | --- | --- |
|  | **FEV_1_**  **% predicted** | **FRC**  **% predicted** | **BMI**  **kg/m²** | **Diastolic BP**  **mm Hg** |
| **LV mass normalized, g/m^2^** | 0.027  [-0.084; 0.138] | -0.069* [-0.138; 0.000] | 0.934* [0.535; 1.334] | -0.010  [-0.196; 0.177] |
| **RR interval, ms** | 1.678*  [1.242; 2.114] | -0.280* [-0.553; -0.007] | -2.649* [-4.221; -1.077] | -1.805* [-2.540; -1.070] |
| **QT duration, ms** | 0.230*  [0.135; 0.325] | -0.099* [-0.158; -0.039] | -0.315  [-0.659; 0.029] | -0.328*  [-0.489; -0.168] |
| **P axis, degree** | -0.187*  [-0.266; -0.108] | 0.129* [0.080; 0.179] | -0.663* [-0.948; -0.378] | -0.155* [-0.288; -0.022] |
| **QRS axis, degree** | -0.164*  [-0.298; -0.030] | 0.192* [0.108; 0.276] | -0.585*  [-1.067; -0.102] | -0.244* [-0.470; -0.019] |
| **T axis, degree** | -0.198*  [-0.269; -0.126] | 0.135*  [0.090; 0.180] | -0.732*  [-0.990; -0.474] | -0.124 [-0.245; 0.004] |

Non-standardized coefficients from multivariate multiple linear regression analysis and 95% confidence intervals for the set of echocardiographic and ECG parameters analyzed in GOLD 0-4, with the covariates FEV_1_ % predicted, FRC % predicted, BMI, and diastolic blood pressure. FEV_1_ = forced expiratory volume in one second, FRC = functional residual capacity; BMI = body-mass index; BP = blood pressure. In this analysis, the confounders sex, age and medication were taken into account by using the appropriately adjusted values. Confidence intervals corresponding to a statistically significant (p<0.05) dependence are marked with an asterisk.

**Table E3. Results of the structural equation model (SEM)**

**Regression weights**

| **Directed Relationships** | | | **Estimate** | **S.E.** | **C.R.** | **p value** |
| --- | --- | --- | --- | --- | --- | --- |
| RR interval | **←** | FEV_1_ | 1.931 | 0.182 | 10.582 | < 0.001 |
| ECG axes | **←** | FEV_1_ | -0.199 | 0.037 | -5.362 | < 0.001 |
| LV mass | **←** | FRC | -0.079 | 0.029 | -2.706 | 0.007 |
| ECG axes | **←** | FRC | 0.154 | 0.024 | 6.405 | < 0.001 |
| LV mass | **←** | BMI | 0.930 | 0.203 | 4.582 | < 0.001 |
| RR interval | **←** | BMI | -2.161 | 0.764 | -2.83 | 0.005 |
| ECG axes | **←** | BMI | -0.816 | 0.136 | -6.015 | < 0.001 |
| RR interval | **←** | Diast. BP | -1.803 | 0.373 | -4.831 | < 0.001 |
| ECG axes | **←** | Diast. BP | -0.200 | 0.058 | -3.426 | < 0.001 |
| QT duration | **←** | LV mass | 0.065 | 0.017 | 3.915 | < 0.001 |
| QT duration | **←** | RR interval | 0.161 | 0.004 | 39.793 | < 0.001 |
| ECG_axes | **←** | QT duration | -0.081 | 0.021 | -3.901 | < 0.001 |
| P axis | **←** | ECG axes | 0.800 | 0.081 | 9.828 | < 0.001 |
| QRS axis | **←** | ECG axes | 1.000 | - | - | reference |
| T axis | **←** | ECG axes | 0.882 | 0.086 | 10.283 | < 0.001 |
| **Covariances** | | | | | | |
| **Undirected Relationships** | | | **Estimate** | **S.E.** | **C.R.** | **p value** |
| FRC | ↔ | FEV_1_ | -409.786 | 23.568 | -17.387 | < 0.001 |
| FEV_1_ | ↔ | BMI | 16.521 | 2.957 | 5.588 | < 0.001 |
| FRC | ↔ | BMI | -56.895 | 5.136 | -11.077 | < 0.001 |

Regression weights and covariances for the SEM in Figure 3. S.E. = standard error, C.R. = critical ratio (estimate/S.E.).
p-value the type-I error according to the Wald statistic. The estimates are based on maximum likelihood estimation. The same pattern of significant estimates (with slightly altered numerical values) was obtained with generalized least squares estimation and with asymptotically distribution-free estimation. FEV_1_ and FRC were evaluated as % predicted and the dimensions of all variables are as in Table 1 and 2. BMI = body mass index, Diast. BP = diastolic blood pressure, RR interval = interval between adjacent R peaks of the QRS complex, QT duration = interval including de- and repolarization as defined by standard.

## Sensitivity analyses

The SEM was obtained after all parameters had been adjusted for sex, age and heart rate-lowering medication. The same structure could be obtained when using unadjusted values, but this resulted in lower goodness of fit. Moreover, when using values adjusted only for sex and age but not medication, the same structure could be validated. When medication was introduced as a further variable in terms of a binary predictor at the “top level” of lung function, BMI and diastolic blood pressure (Figure 3), the structure was still fully maintained, with medication showing a significant relationship to LV mass and RR interval but not to other variables. To account for potential differential effects of lung function on the QRS axis (Figure 1 and 2), addition relationships from FRC % predicted and/or FEV_1_ % predicted to the QRS axis were tentatively introduced. These were not statistically significant, and did not improve the overall model fit. Use of FEV_1_ and FRC z-scores instead of adjusted values % predicted yielded concordant results in the final model.

For the present analysis, 1195 patients with full and plausible data on lung function, echocardiography and ECG out of 2741 COSYCONET patients were analyzed. One might argue that there could be a selection bias. Therefore, the SEM analysis was re-run without echocardiography-derived LV mass as intermediate variable. Omission of this selection criterion resulted in a number 2015 eligible patients. The formerly identified indirect influence of FRC % predicted on QT duration via LV mass (Figure 3) now changed into a direct influence on QT duration, while the influence of BMI on QT duration, as formerly mediated via LV mass, was no more significant. All other relations of the SEM remained unaffected regarding their statistical significance, in particular those referring to the ECG axes (n=2015; CFI 0.989; RMSEA 0.034; 90%CI 0.025; 0.043). Essentially the same result was obtained when omitting LV mass as variable in the reduced data set (n=1195; CFI 0.994; RMSEA 0.024; 90%CI 0.008; 0.038). These observations do not suggest a selection bias.

# Discussion

We adjusted all variables for the effects of sex and age, as well as medication, since otherwise these influencing factors would have impacted the correlations in the SEM (see Figure 3 and Table E2). To explicitly evaluate the effect of medication, we performed a sensitivity analysis in the SEM using variables that had been adjusted for sex and age only. Medication was then introduced by addition of a separate variable at the level of the top predictors. This binary variable comprised the presence vs. absence of heart-rate lowering medication. Within the SEM structure, the medication variable was significantly associated only with the RR interval and with LV mass. In particular there were no direct effects on the QT duration or ECG axes, which apparently were completely explained through indirect effects mediated via RR interval and LV size. These results illustrated the potential of SEM to simplify multiple relationships by attributing them to direct and indirect effects.

# References

1. Karch A, Vogelmeier C, Welte T, Bals R, Kauczor HU, Biederer J, Heinrich J, Schulz H, Glaser S, Holle R, et al: **The German COPD cohort COSYCONET: Aims, methods and descriptive analysis of the study population at baseline.** *Respir Med* 2016, **114:**27-37.

2. Vogelmeier CF, Criner GJ, Martinez FJ, Anzueto A, Barnes PJ, Bourbeau J, Celli BR, Chen R, Decramer M, Fabbri LM, et al: **Global Strategy for the Diagnosis, Management, and Prevention of Chronic Obstructive Lung Disease 2017 Report: GOLD Executive Summary.** *Eur Respir J* 2017, **49**.

3. Alter P, Jorres RA, Watz H, Welte T, Glaser S, Schulz H, Bals R, Karch A, Wouters EFM, Vestbo J, et al: **Left ventricular volume and wall stress are linked to lung function impairment in COPD.** *International Journal of Cardiology* 2018, **261:**172-178.

4. Alter P, Watz H, Kahnert K, Pfeifer M, Randerath WJ, Andreas S, Waschki B, Kleibrink BE, Welte T, Bals R, et al: **Airway obstruction and lung hyperinflation in COPD are linked to an impaired left ventricular diastolic filling.** *Respir Med* 2018, **137:**14-22.

5. Jung JY, Park SK, Oh CM, Kang JG, Choi JM, Ryoo JH, Lee JH: **The influence of prehypertension, controlled and uncontrolled hypertension on left ventricular diastolic function and structure in the general Korean population.** *Hypertens Res* 2017.
